# Examining intraindividual variability of urinary gluten immunogenic peptides compared to the lactulose-mannitol ratio in healthy volunteers: implications for clinical assessment of intestinal permeability

**DOI:** 10.3389/fimmu.2025.1633500

**Published:** 2025-09-04

**Authors:** Raquel Rodríguez-Ramírez, María Auxiliadora Fernández Peralbo, Ángel Cebolla, Carolina Sousa

**Affiliations:** ^1^ Research and Development Department, Biomedal S.L., Seville, Spain; ^2^ Inorganic Chemistry Department, Faculty of Science, University of Granada, Granada, Spain; ^3^ Department of Microbiology and Parasitology, Faculty of Pharmacy, University of Seville, Seville, Spain

**Keywords:** intestinal permeability, urine, gluten immunogenic peptides, lactulose, mannitol

## Abstract

**Introduction:**

The intestinal barrier plays a crucial role in preventing the translocation of harmful substances. Intestinal permeability is commonly assessed using the lactulose-mannitol ratio (LMR) test, which measures absorption of non-metabolizable sugars. However, it does not reflect permeability to larger, potentially antigenic molecules such as proteins. Urinary gluten immunogenic peptides (u-GIP), derived from gluten, have emerged as promising biomarkers, showing sensitivity to mucosal disruption. This study compared the intraindividual variability of u-GIP and LMR under fasting conditions in healthy volunteers to assess the consistency and reproducibility of both methods for evaluating intestinal barrier function.

**Methods:**

Twelve healthy adult volunteers underwent a controlled dietary protocol and repeated intestinal permeability testing on three separate days. After a fasting period, each participant ingested gluten, lactulose and mannitol. Urine samples were collected at 0–6 and 0–15-hour intervals. GIP levels were assessed using a lateral flow immunoassay, while lactulose and mannitol were quantified via ion chromatography. Intraindividual variability was evaluated using coefficients of variation (CV) and intraclass correlation coefficients (ICC) and the results were compared with reference ranges.

**Results:**

Excretion patterns for u-GIP, lactulose and mannitol were generally similar, with detection within the first 3 hours and peaks around 4–5 hours. u-GIP consistently exhibited lower intraindividual variability than the traditional LMR. The CV for u-GIP excretion remained within an acceptable range (~20%) and its ICC reached 0.750 in the 0–15-hour interval, indicating excellent reproducibility. In contrast, the LMR showed higher CVs and poor ICC values, which only improved modestly after outlier exclusion. GIP measurements consistently fell within established reference ranges across test repetitions, further supporting their stability.

**Discussion:**

u-GIP demonstrated lower intraindividual variability and higher consistency than LMR, indicating its potential as a robust and reliable marker for assessing intestinal permeability. Unlike LMR, GIP showed better reproducibility across days and minimal influence from dietary fluctuations. Its advantages include direct clinical relevance as an immunogenic dietary protein marker, reduced physiological variability and non-invasive methods. These features highlight u-GIP promising potential for future clinical and research applications. However, further studies are needed to validate its effectiveness specifically in patients with altered intestinal permeability.

## Introduction

The intestine serves as the largest interface between the body and the external environment. Thus, maintaining the intestinal barrier function is crucial for preventing the translocation of luminal substances and pathogens into the internal milieu. This barrier is a dynamic system that depends on the integrity of the intestinal epithelium (1) and is influenced by factors such as the gut microbiome composition, hormones, dietary components, inflammatory mediators and the enteric nervous system, which regulates intestinal secretion and motility independently of the brain (2). Under physiological conditions, the intestinal barrier must maintain a balance between selective nutrient absorption and protection against pathogenic entry. Compromised intestinal barrier integrity can lead to a condition referred to as “leaky gut”, which permits an uncontrolled movement of bacterial components and harmful substances into the systemic circulation. This can result in systemic inflammation and contribute to a wide range of diseases, such as inflammatory bowel disease, type 1 diabetes, non-alcoholic liver disease and various autoimmune conditions (3).

The lactulose-mannitol ratio (LMR) test serves as a reference method for the assessment of intestinal permeability (4). It involves the oral administration of lactulose and mannitol, two sugars that are absorbed differently: lactulose is absorbed paracellularly, whereas mannitol is absorbed transcellularly. Under conditions of increased intestinal permeability, lactulose appears at higher levels in the urine, while mannitol absorption is reduced in diseases associated with villous atrophy, both contributing to an elevated LMR (5). The simplicity and non-invasive nature of the LMR test make it an attractive tool for evaluating intestinal barrier function (6). Nevertheless, the LMR test has several limitations, among which two principal methodological challenges stand out. First, urine-collection windows vary widely, most studies use between 2 h and 5–6 h and the length of this window has a marked effect on sugar recovery and the resulting LMR (7, 8). Mannitol is absorbed and excreted more rapidly whereas lactulose peaks later; therefore, short collections risk missing late lactulose excretion and artificially lowering the calculated ratio (8). Second, there is no universally accepted protocol for the LMR test and cut-off values deemed “normal” or “abnormal” differ by clinical context, which hampers reproducibility and interpretation (8–10). These methodological discrepancies underscore the need for harmonized testing procedures to ensure accuracy and consistency in assessing intestinal permeability (11). Additionally, the analytical quantification of lactulose and mannitol in urine typically requires high-performance liquid chromatography (HPLC) or liquid chromatography coupled to mass spectrometry (LC-MS), which are technically demanding, time-consuming and require specialized equipment and expertise (7). Furthermore, the LMR test only determines the potential permeability of small molecules such as sugars and does not assess the absorption of larger macromolecules or antigenic proteins that may be relevant in inflammatory and autoimmune diseases. Lactulose is also used as a prebiotic that may affect the microbiota and promote diarrhea in certain individuals (12). Mannitol is a component of multiple foods that might affect the quantification of the probe, as we observed in our first study (13).

In a previous study (13), we investigated the combined use of urine gluten immunogenic peptides (u-GIP) and LMR to explore intestinal permeability. GIP are peptides derived from partially digested gluten and their presence in urine indicates their ability to cross the intestinal barrier and be excreted by the kidneys. Given the size of detectable GIP, they are likely to traverse the paracellular pathway, which is often compromised in cases of increased intestinal permeability, they offer a valuable model for studying the absorption of biologically active macromolecules. We examined the kinetics of u-GIP and LMR after the simultaneous consumption of gluten, lactulose and mannitol. Our results indicated that while GIP and lactulose showed similar excretion patterns, GIP reached their excretion peak earlier than lactulose or mannitol. Additionally, the moderate correlation between LMR and u-GIP suggests potentially shared characteristics of the permeability pathways. Notably, this study revealed that extending the urine collection beyond the standard 6-hour period used in most LMR tests (14) may provide more comprehensive data for the selection of the best protocol to minimize variability in the analysis, as our findings suggest that valuable information related to the kinetics of absorption may be lost with shorter collection times.

Assessing intraindividual variability in the analysis of intestinal permeability is crucial for accurately understanding and interpreting the dynamics of intestinal barrier function. Variability in permeability measurements can arise from numerous individual factors, including dietary intake, gastrointestinal motility, individual differences in the gut microbiota and gastrointestinal fluid composition. Evaluating the potential variability within the same individual is the key to distinguishing between normal physiological fluctuations, pathological changes in intestinal permeability and patient evolution. Our study aimed to address this gap by examining the intraindividual variability in intestinal permeability through repeated testing of both GIP and the LMR. By performing these tests several times per volunteer in different weeks, we aimed to assess whether u-GIP as a potential biomarker offers a more consistent and reliable measure of intestinal barrier integrity than the LMR alone. An additional advantage of u-GIP is that they can be easily detected using a non-invasive lateral flow immunoassay (LFIA), facilitating rapid and convenient assessment of intestinal permeability in clinical and field settings. This approach provides insights into the stability and reliability of these biomarkers, potentially improving their use in both clinical and research contexts.

## Materials and methods

### Study population

Twelve healthy volunteers were included based on the following criteria (1): age >18 years (2); absence of celiac disease (CD), non-celiac gluten sensitivity, food allergies, food intolerances, gastrointestinal diseases, metabolic disorders, cardiovascular diseases, or other systemic conditions that could affect intestinal permeability (3); willingness to adhere to a strict diet regimen; and (4) capability to collect daily urine samples.

To rule out CD and gluten-related disorders, all participants completed a symptom-based questionnaire (15) and underwent a CeliacDetect^®^ rapid test (Biomedal, Seville, Spain), which detects anti-tTG IgA antibodies in capillary blood.

Exclusion criteria included (1): presence of concurrent pathologies.

This study was conducted in accordance with the guidelines of the Declaration of Helsinki. All participants provided written informed consent, and the study was approved by the local ethics committee (no. 1308-N-23).

### Study design

**Figure 1:**
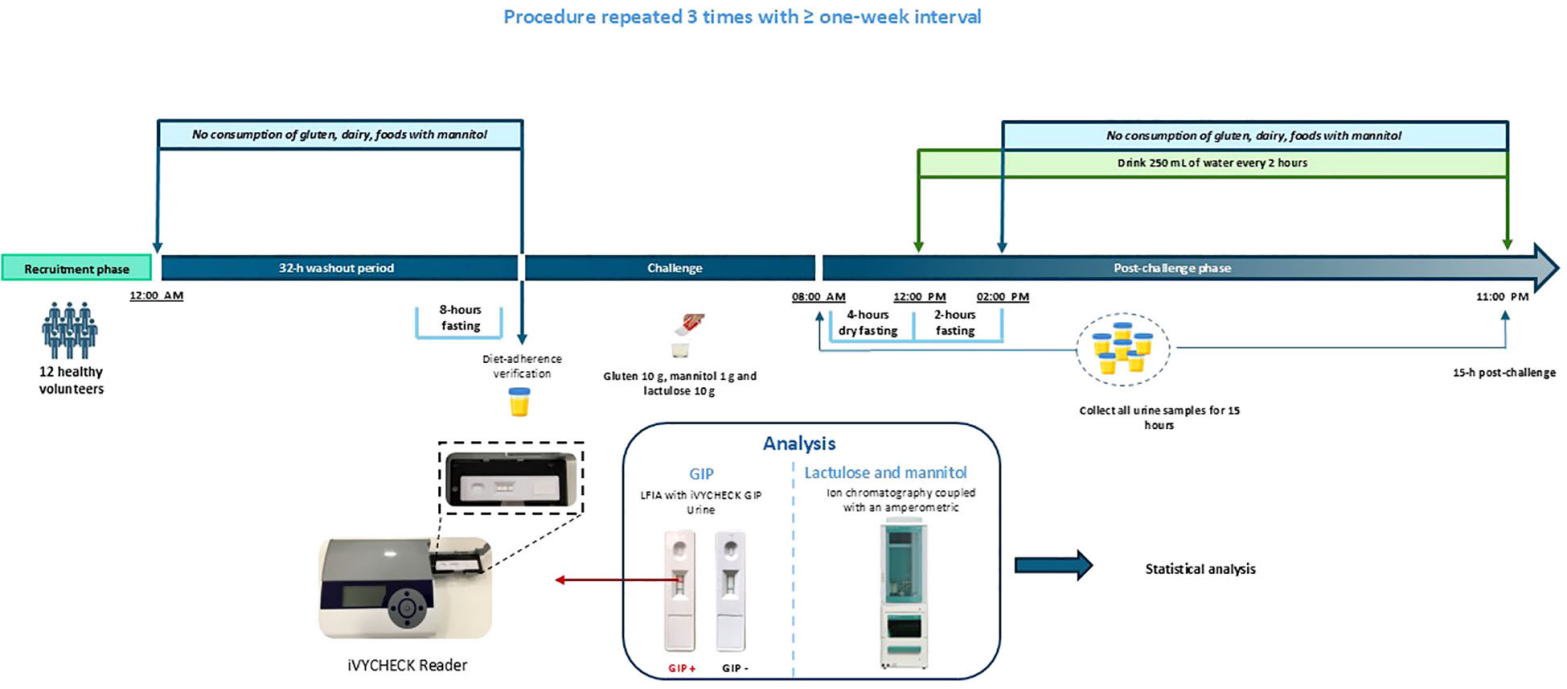
Study timeline illustrating fasting periods, oral challenge administration, liquid intake, and urine sample collection. Twelve healthy volunteers completed the protocol on three separate occasions, with washout periods of at least one week between sessions. Each visit included a 32-hour washout, followed by an 8-hour fasting period prior to arrival and an additional 6-hour fasting period onsite before the challenge. Dietary restrictions (gluten-, dairy-, and mannitol-free) were maintained throughout the study. The oral challenge consisted of 10 g gluten, 10 g lactulose, and 1 g mannitol. Water intake began 4 hours after the challenge, with 250 mL consumed every 2 hours thereafter, under continued dietary restrictions. Urine samples were collected continuously for 15 hours post-challenge (0–15 h; typically, 08:00 AM–11:00 PM). GIP, gluten immunogenic peptides; LFIA, lateral flow immunoassay.

The study consisted of two stages: a washout period and an intake/collection period (**Figure 1**). The washout period lasted for 32 hours (including a final 8-hour fasting period) during which volunteers adhered to a gluten-free diet and abstained from dairy and foods with high sorbitol and/or mannitol content. Prior to ingestion, the volunteers provided urine samples to confirm the absence of the target compounds. Gluten (10 g), lactulose (10 g) and mannitol (1 g) were ingested after a period of eight hours fasting. Participants fasted for the initial 4 hours after ingestion and subsequently commenced the scheduled liquid intake (250 mL every 2 hours). At the 6-hours mark, the participants began a restricted (gluten-free, low-mannitol) diet. The study concluded 15 hours after the ingestion of the compounds.

Throughout the study, diet adherence and fluid intake were assessed using a food recall questionnaire and the participants recorded their daily food consumption details. A schematic of the study timeline is shown in **Figure 1**.

Twelve individuals (six [50%] females and six [50%] males) completed the study. The median age of the participants was 30 years (IQR 19 years). None of the participants had been diagnosed with any relevant diseases.

This procedure was repeated thrice, with a minimum interval of one week between each repetition.

### Administration of compounds

The compounds comprised 10 g of gluten powder and 1 g of mannitol (Aurora Intelligent Nutrition, Sevilla, Spain) provided together in a single sachet and an oral solution sachet of lactulose (Duphalac™, Abbott Laboratories, S.A., Madrid, Spain). Gluten and mannitol were mixed in 125 mL of water with vigorous shaking. After ingestion, 125 mL of water was added to ensure a complete suspension of the remaining powder.

### Urine collection

Detailed instructions were provided to all participants at the start of the study. The subjects were equipped with all necessary materials for urine collection, including plastic screw-capped containers, labels, cool bags, isothermal boxes and cool packs. The participants were instructed to collect the entire urine sample from each micturition, noting the date and time of collection. All urine samples were preserved in isothermal boxes with cool packs at 4–8^°^C and deposited within 48 hours of collection. Urinary GIP levels were confirmed to remain stable for up to 78 hours when stored at 4–8°C, based on testing of negative, borderline, and positive samples across multiple time points. The samples were then frozen at -20°C until processing. The GIP concentration in the urine remained stable throughout the freeze-thaw process.

### Urine analysis

The volume of each urine sample was recorded. When multiple containers were required for the same urination, the samples were mixed and homogenized. Additionally, some mixed samples were analyzed at 0–6-hours and 0–15-hours intervals. To prepare these mixtures, 10% of the volume of each container was used. To prevent bacterial growth, 100 µL of 1% chlorhexidine diacetate was added. Aliquots of 1 mL were stored at -20°C until analysis.

#### GIP analysis

Qualitative analysis of GIP in urine was conducted using a LFIA with iVYCHECK GIP Urine (Biomedal S.L., Seville, Spain), following the manufacturer’s guidelines. Thawed urine samples were homogenized and mixed with a conditioning solution. Subsequently, 100 µL of the mixture was added to the immunochromatographic cassette and visual interpretation of results occurred after 30 minutes. A positive outcome was determined if the test line exhibited a red color accompanied by a green color on the control line. A negative result was confirmed when only the control line displayed a green color. The limit of detection determined by visual inspection was 2.50 ng GIP/mL urine. GIP concentration in urine was also assessed on the immunochromatographic strips using the iVYCHECK Reader (Biomedal S.L., Seville, Spain). Reader calibration was performed prior to urine analysis with α-gliadin 33-mer peptide as a standard. The dynamic range established for this method was 3.12–25 ng GIP/mL urine. The results were expressed in “ng GIP per mL of urine”. Each mixed sample (0–6-hours and 0–15-hours intervals) was subjected to duplicate experiments. The results presented in this paper are expressed in absolute amounts, as they were calculated by considering both the concentration and volume of each urine sample.

#### Lactulose/mannitol analysis

Lactulose and mannitol analytes were determined using ion chromatography coupled with an amperometric detector (IC-PAD), with a linear range of 10–1500 mg/L. The Ion Chromatography (IC) system used a 930 Compact IC Flex (Metrohm, Herisau, Switzerland) equipped with a Metrosep Carb 2 column (5 µm, 4 x 150 mm) (Metrohm, Herisau, Switzerland). Chromatographic separation was performed using a mobile phase of 300 mM NaOH and 1 mM sodium acetate, operating in isocratic mode with a constant flow rate of 0.5 mL/min. The injection volume was 5 µL; the oven temperature was maintained at 30°C, whereas the column compartment was set to 6°C.

Detection was carried out using an amperometric detector operating at a temperature of 35°C. The electrochemical cell was equipped with a Pd reference electrode and a gold working electrode.

The aliquots were thawed and agitated for one minute using a vortex mixer. Subsequently, they were centrifuged for 5 minutes at 5,000 g to remove sediments. Fifty microliters of the supernatant were collected and brought to a final volume of 1 mL with water. The final dilution ratio was 1:20 (v/v).

The results presented herein are expressed as absolute amounts, as they were calculated by considering both the concentration and the volume of each urine sample.

### Statistical analysis

Quantitative variables results were presented using both the mean (SD) and median (IQR or range), whereas categorical variables were expressed as absolute (N) and cumulative (%) frequencies. The LMR values were then multiplied by 100. Interquartile tests were performed using RStudio version 2022.02.3 + 492 (RStudio, Inc., Boston, MA, USA) and correlation tests were conducted using Microsoft Excel (Version 2401, Microsoft Corporation, Redmond, WA, USA). Intraclass correlation coefficients (ICCs) were calculated using Python version 3.10.12 (Python Software Foundation) in Google Colab with the `pandas` and `pingouin` libraries. A random effects analysis of variance was used to estimate the ICC and its 95% confidence interval (95% CI) considering the three repetitions of the test for each volunteer. ICC values were interpreted as follows: <0.4 as “poor” correlation; 0.4–0.75, “fair” to “good” correlation; >0.75, “excellent” correlation (16).

## Results

### Dietary compliance monitoring and standardized urine collection protocol

To ensure reliable assessment of intestinal permeability, participants adhered to a controlled dietary protocol documented through detailed dietary records. Following an eight-hour fasting period, each participant ingested a standardized solution containing gluten, lactulose and mannitol, as specified in the study protocol. Urine samples were collected according to the designated time intervals (0–6-hours and 0–15-hours) post-ingestion. Despite these measures, one participant (Volunteer 10) displayed an aberrant permeability profile that deviated significantly from the expected post-intervention values. This pattern suggested potential noncompliance or incomplete ingestion of the test compounds and their data were therefore excluded from further analysis.

A total of 215 urine samples were collected from all participants. It is essential to emphasize the meticulous attention paid to the pooling process in the two periods of estimation periods to maintain proportionality and timing. Most samples followed the study protocol precisely, but some volunteers showed slight deviations beyond the specified collection time during this interval. The deviation range in the 0–15-hour interval, was a maximum of ±10 minutes, whereas deviations in the 0–6-hour interval, reached up to a maximum of 2 hours. Approximately 40% of the samples corresponding to the 0–6-hour interval exhibited variations in collection timing. In most cases, this was due either to the absence of a sample within the defined timeframe or to the inclusion of a sample collected shortly after the 6-hour mark, when no intermediate sample was available.

Considering these exceptions, the statistical analyses provided valuable insights into the mixing procedures. For the 0–6-hours interval, the median number of urine samples used for mixing was 2 (IQR 1), with a mean pooled six-hourly urine volume of 356 mL ± 173 mL. In contrast, for the 0–15-hour interval, the median number of urine samples employed for pooling was 6 (IQR 2.25), resulting in an average total volume of 954 mL ± 439 mL.

Excluding baseline urination, the average number of urinations per participant during the 15 hours period was determined to be 6 ± 2. Importantly, baseline urinations showed non-detectable levels of u-GIP and lactulose, indicating successful adherence to the pre-study dietary requirements. However, an average baseline of 39 ± 101 ppm of mannitol was detected in these samples, highlighting the challenges of adhering to a mannitol-free diet, despite recommendations to exclude foods rich in mannitol.

### Urinary excretion kinetics of intestinal permeability markers and interindividual variability

The excretion kinetics of u-GIP following ingestion were analyzed, revealing distinct patterns among the participants. On average, the first urine sample in which GIP were detectable was excreted at 3 hours 30 minutes after ingestion. For most volunteers, this corresponded to their first post-ingestion urination. The average time at which peak excretion occurred was 4 hours 45 minutes, with an average excreted amount of 3.30 µg (SD 1.32) at this peak. This value was calculated as the mean of the maximum excreted amounts observed at the individual peak excretion times across all volunteers. Detectable GIP excretion continued up for an average of 9 hours 55 minutes after ingestion, with the first undetectable urine sample appearing at 12 hours 36 minutes after ingestion on average. Notably, a double peak in excretion was observed on one day for one volunteer, which may have been related to the end of the fasting period in the study, suggesting two zones of different intestinal permeability. The mean coefficient of variation (CV) was 29% for the time to peak excretion, considering 3 days for each volunteer and 23% for the maximum GIP excreted in a single urine sample. Despite this intraindividual variability, the ANOVA showed no statistically significant differences (*p* = 0.717) between days.

Analysis of lactulose excretion kinetics revealed distinct temporal patterns among the participants. The first urine sample with detectable lactulose levels was typically excreted approximately at 3 hours 11 minutes after ingestion, which closely aligned with the timing of u-GIP excretion. The first detectable lactulose level was observed in the first urine sample after ingestion. The average peak excretion time for lactulose was 5 hours 19 minutes, with a mean peak excretion of 21.4 mg (SD 9.21). Lactulose excretion continued for an average of 9 hours 47 minutes after ingestion, with the first urine sample with undetectable levels appearing, at 12 hours 22 minutes after ingestion on average. Notably, lactulose did not reach undetectable levels in three volunteers on all study days, suggesting possible distal absorption of the molecule. Additionally, a double peak in excretion was observed in three volunteers: one volunteer experienced this phenomenon on all three study days, whereas the other two experienced it on two study days. This suggests that individual reproducible characteristics affect the lactulose absorption and excretion patterns. The mean CV was 24% for the time to peak excretion, considering three days for each volunteer and 38% for the maximum amount of lactulose excreted in a single urine sample. Despite this intraindividual variability, the ANOVA showed no statistically significant differences (*p* = 0.217) between days.

As mentioned previously, all baseline urine samples exhibited quantifiable mannitol values. The average peak excretion time was 4 hours 58 minutes, with a mean peak excretion of 124 mg (SD 107). No double excretion peaks were simultaneously observed for mannitol in any individual across all the three study days. However, double peaks were detected on at least one of the three days in 37% of the volunteers. The absence of mannitol in urine samples was not observed in any of the volunteers during the study. The mean CV was 22% for the time to peak excretion, considering three days for each volunteer and 30% for the maximum amount of mannitol excreted in a single urine sample. Despite this intraindividual variability, ANOVA showed no statistically significant differences (*p* = 0.948) between days.

A summary of the excretion kinetics and variability measures for u-GIP, lactulose and mannitol across participants is presented in **Table 1**.

**Table 1:** Summary of excretion kinetics and interindividual variability for urinary GIP, lactulose, and mannitol across study participants.

Parameter	GIP	Lactulose	Mannitol
Initial detectable urine sample (time)/urine sample	3 hours 30 minutes/first urine after ingestion	3 hours 11 minutes/first urine after ingestion	Baseline
Average time peak excretion (time)	4 hours 45 minutes	5 hours 19 minutes	4 hours 58 minutes
Average amount in peak excretion	3.30 µg (SD 1.32)	21.4 mg (SD 9.21)	124 mg (SD 107)
Last detectable parameter excretion (time)	9 hours 55 minutes	9 hours 47 minutes	Always detectable
First urine sample with undetectable level (time)	12 hours 36 minutes	12 hours 22 minutes	Always detectable
Double peak excretion	1 volunteer (1/3 days)	3 volunteers (3/3 days, 2/3 days, 2/3 days)	37% volunteers (at least 1/3 days)
Time peak excretion CV (%)	29%	24%	22%
Maximum amount excreted in a single urine CV (%)	23%	38%	30%
ANOVA test (differences between days)	No (*p* = 0.717)	No (*p* = 0.217)	No (*p* = 0.948)

CV, coefficient of variation; GIP, gluten immunogenic peptides; SD, standard deviation; ANOVA, analysis of variance.

### Outliers in urinary excretion of permeability markers identified via interquartile range to assess interindividual variability

In order to evaluate the reliability of the markers and detect unusual physiological responses that could compromise the interpretation of intestinal permeability, a study of atypical cases was conducted with the volunteers to determine if any participant demonstrated outlier values in the assessed parameters [LMR, u-GIP (µg), mannitol (mg), lactulose (mg)] at different time intervals. These atypical cases results are summarized in **Table 2**, which shows that lactulose was the parameter with the highest number of outlier values. The outliers were identified using the Interquartile Range (IQR) method. Using this approach, the first quartile (Q1), representing the value below which 25% of the data fell and the third quartile (Q3), representing the value below which 75% of the data fell, were calculated. Any values falling below Q1 – 1.5 × IQR or above Q3 + 1.5 × IQR were classified as outliers. This method was selected to ensure the detection of significant deviations from the central tendency of the data, which could indicate biological variability, measurement inconsistencies, or abnormal responses to the ingested compounds.

**Table 2:** Outlier values for selected urinary parameters measured at 0–6-hour and 0–15-hour collection intervals.

Parameter	Interval	Q1	Q3	Lower range	Upper range	Outlier
LMR	0–6-hours	1.23	1,.78	0.41	2.80	3.48-Vol 3. Day 1
						3.27-Vol 12. Day 1
u-GIP (µg)	0–6-hours	3.54	6.04	0.00	9.80	11.15 mg-Vol 1. Day 1
Lac (mg)	0–6-hours	25.10	36.78	7.57	54.31	67.56 mg-Vol 3. Day 1
Lac (mg)	0–15-hours	38.44	57.98	9.12	87.29	88.86 mg-Vol 3. Day 1
						94.50 mg- Vol 9. Day 2
Man (mg)	0–6-hours	115.65	158.48	51.40	222.72	229.97 mg-Vol 8. Day 2

LMR, lactulose-mannitol ratio; GIP, gluten immunogenic peptides; Lac, lactulose; Man, mannitol; Q1, first quartile; Q3, third quartile; Vol, Volunteer.

### Intraindividual variability and reliability of permeability markers assessed by reference ranges, CV and ICC across urine collection intervals

The intraindividual variability and consistency of the key parameters determined in this study were evaluated to assess the quantitation of urine GIP as a potential reliable marker of individual permeability. This analysis was conducted in a cohort of 12 healthy volunteers, which is considered sufficient for intraindividual comparisons, as each participant underwent repeated measurements under standardized conditions. First, we compared the results obtained with reference ranges established in a previous study involving 15 volunteers, who completed the same protocol at a single time point. These values were considered applicable as both studies were conducted under identical conditions, including compound doses, dietary restrictions and urine collection procedures and no other reference data under such standardized conditions are available. Next, we calculated the CV for each individual, considering each parameter across two distinct intervals (namely, 0–6-hours and 0–15-hours intervals) that could be used in the protocol to assess the variability of the parameters over different days. Finally, we evaluated the consistency of the measurements using the ICC, which indicated the reliability of the measurements across these two-time intervals.

**Figure 2:**
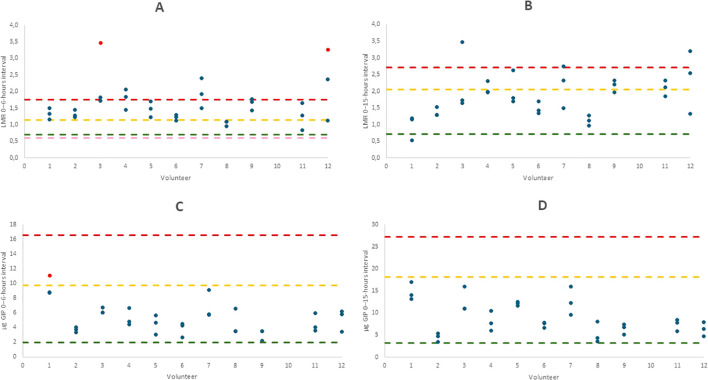
Graphs showing the comparison of different parameters across volunteers: LMR 0–6-hours interval **(A)**, LMR 0–15-hours interval **(B)**, u-GIP 0–6-hours interval **(C)** and u-GIP 0–15-hours interval **(D)**. Results are displayed per volunteer, with outliers highlighted in red. Reference ranges from the previous study were established with and without considering outliers. Maximum ranges are indicated in yellow, minimum ranges in green. The maximum range including outliers is shown in red and the minimum range with outliers, if applicable, is shown in pink. LMR, lactulose-mannitol ratio; u-GIP, urine gluten immunogenic peptides.

A comparison was made between the results obtained in this study and those of a previous study involving 15 volunteers who completed the same protocol once. Reference excretion ranges were established based on the results of a previous study across two distinct time intervals, as shown in **Table 3**. The outliers were identified and marked for clarity, as shown in **Figure 2**. In (A), which shows the LMR parameter at the 0–6-hour interval, there is greater variability among volunteers and several values outside the reference range, including outliers—indicating lower consistency during this period. In contrast, LMR at 15 hours (B) displays reduced dispersion and values largely within the established ranges. For u-GIP, the 0–6-hour interval (C) includes a single outlier, while the 0–15-hour interval (D) shows no values outside the range, suggesting greater stability and lower interindividual variability in this marker over time. Reference ranges are shown both with and without outliers to adequately reflect physiological variability in healthy individuals. Ranges excluding outliers allow for a stricter assessment of values, while those including them provide a broader and more realistic context, helping to avoid misinterpretation of values that may fall within normal interindividual variation.

**Table 3:** Lower and upper bounds of urinary excretion intervals for GIP and LMR in the 0–6-hour and 2–15-hour periods, presented with and without outliers.

Parameter	Interval	Lower bound (without outliers)	Upper bound (without outliers)	Lower bound (with outliers)	Upper bound (with outliers)
u-GIP (µg)	0–6-hours	1.96	9.71	1.96	16.54
	0–15-hours	3.17	18.18	3.17	27.19
LMR	0–6-hours	0.70	1.14	0.60	1.77
	0–15-hours	0.71	2.05	0.71	2.71

LMR, lactulose-mannitol ratio; u-GIP, gluten immunogenic peptides excreted.

**Figure 3:**
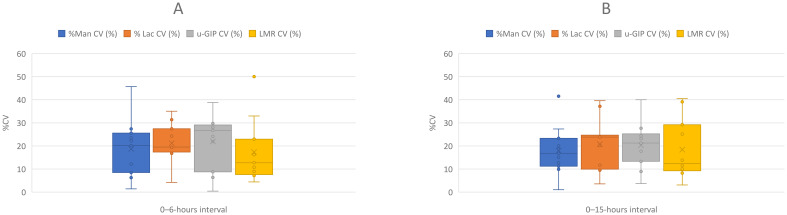
Box-and-whisker plots comparing the coefficient of variation (%CV) for different parameters across two urine collection intervals: **(A)** (0–6-hour interval) and **(B)** (0–15-hour interval). The parameters compared include %Man CV (blue), %Lac CV (orange), u-GIP CV (gray) and LMR CV (yellow). The plots display intraindividual variability for each parameter, with outliers represented by dots. Whiskers indicate the data range without outliers, while boxes represent the interquartile range (IQR). LMR, lactulose-mannitol ratio; u-GIP, urine gluten immunogenic peptides: Lac, lactulose; Man, mannitol.

Additionally, in this study, each volunteer underwent the protocol three times, with four parameters [LMR, mannitol (mg), lactulose (mg) and u-GIP (µg)] measured in each instance. The CV was calculated across two distinct time intervals (namely, 0–6-hour and 0–15-hour intervals). These CVs represent the intraindividual variability by considering the results obtained over the three study days for each parameter. Outliers were excluded from the analysis to avoid inflating variability estimates. Given the CV sensitivity to extreme values, this approach provides a more accurate reflection of physiological consistency. The findings are presented in **Figure 3**, which displays the range of CVs for each parameter as box plots, highlighting the spread and central tendency of intraindividual variability.

The ICC is a statistical metric used to assess the reliability of measurements by evaluating the consistency or agreement between measurements obtained under similar conditions. It measures the proportion of the total variability attributed to differences between groups or conditions relative to the total variability observed. The ICC was calculated for each parameter across different time intervals, considering both the inclusion and exclusion of outliers. **Table 4** presents these results, including the ICC values, *p*-values and interpretations and provides an interpretation of the ICC values, categorizing them as excellent, good or poor according to Kuzma et al. (17). This analysis offers insights into the reliability and consistency of the measurements obtained throughout the study. The ICC analysis revealed distinct reproducibility patterns for LMR and u-GIP across time intervals.

**Table 4:** ICCs and their interpretations for LMR and GIP, with and without outliers values.

Parameter	Interval	ICC	p-value	Interpretation
u-GIP	0–6-hours	0.614	0.000	Good- Statistically significant
	0–6-hours (without outliers)	0.322	0.052	Poor- Not statistically significant
	0–15-hours	0.750	0.000	Excellent -Statistically significant
LMR	0–6-hours	0.284	0.065	Poor- Not statistically significant
	0–6-hours (without outliers)	0.475	0.012	Poor- Not statistically significant
	0–15-hours	0.340	0.036	Poor- Not statistically significant

LMR, lactulose-mannitol ratio; u-GIP, gluten immunogenic peptides excreted; ICC, intraclass correlation coefficients. Interpretation according to Kuzma et al. (17).

This comprehensive analysis, conducted separately for each parameter, aimed to assess both the variability within individuals and the consistency of the measurements across time, offering insights into the robustness and reliability of the data.

### Analysis of variability in cumulative excretion across two sample collection time frames

The LMR test for assessing intestinal permeability presents two major methodological limitations. The first concerns the duration of urine collection, which varies considerably across studies, typically ranging from 2 to 5–6 hours. This variability significantly influences the recovery rates of the administered sugars and consequently affects the accuracy of the LMR values. Mannitol undergoes faster absorption and renal elimination, while lactulose exhibits delayed excretion; thus, shorter collection periods may fail to capture the later phase of lactulose excretion, leading to an underestimation of the LMR. To investigate this, the excretion percentages of lactulose and mannitol were calculated based on both the ingested and total determined excreted quantities. Subsequently, the percentages of both compounds excreted in the 0–6-hours and 0–15-hours interval mixtures were determined. This approach was employed to determine whether relevant information was missing or whether variability was much higher when urine collection was limited to the first 6 hours. Although the lactulose-mannitol test is typically conducted within this time frame, we aimed to evaluate whether extending the collection period to 15 hours was necessary to capture a higher excretion amount and reduce intraindividual variability. The excretion percentage in the 0–15-hours interval pool was considered as 100% and the proportion of this pool excreted within the 0–6-hours window was then calculated. For lactulose, the mean excretion rate was found to be 69.52% ± 17.54%, while for mannitol, the mean excretion rate was observed to be 77.30% ± 12.29%. Regarding u-GIP excretion, the quantity of u-GIP was utilized for calculations with α-gliadin 33-mer (an immunodominant contributor to gluten immunogenicity) (18) as a standard. Employing the same methodology as with the aforementioned compounds a mean excretion rate of 61.56% ± 17.82% was derived for the 0–6-hours interval mixture, with 100% representing the excretion in the 0–15-hours interval mixture.

The LMR was calculated for each participant in the 0–6-hours and 0–15-hours interval mixtures, yielding means of 1.60 ± 0.59 and 1.83 ± 0.65, respectively.

### Correlation analysis between u-GIP and established intestinal permeability markers (LMR and lactulose)

Given the use of the LMR as a standard for evaluating intestinal permeability, this marker was employed as a reference to assess u-GIP as a potential non-invasive biomarker of gut barrier function. Scatterplots were generated to compare the data from all volunteers across the different parameters. In addition to comparing the LMR and u-GIP quantities at both time intervals, the correlation between the excreted amount of lactulose and u-GIP was also examined independently.

No significant correlation was observed in any of the comparisons when considering the data from the 11 volunteers collectively. This was not unexpected, given that the study group consisted of healthy individuals, which may have contributed to the homogeneity of the results. However, individual variations were noted, with two volunteers showing strong correlations: one displayed a significant correlation between the amount of lactulose and u-GIP excreted in the 0–15-hour interval (R² = 0.997, statistically significant) and another showed a correlation between LMR and u-GIP excretion (R² = 0.996, statistically significant). These findings suggest individual-specific responses because the remaining volunteers did not exhibit similar patterns. It is noteworthy that only three data points per volunteer were used in these comparisons.

## Discussion

This study demonstrated that GIP showed significantly lower intraindividual variability than the LMR, which has been widely used as a marker of intestinal permeability. LMR is based on the differential absorption of two sugars (lactulose and mannitol), while GIP derives from gluten, a dietary protein. This finding suggests that GIP could be a robust biomarker for establishing reference values for normal intestinal function within the same individual. The rapid metabolism of an antigen delivered as a powder with a high surface area for contact with digestive fluids indicates that absorption may be the main differential factor between normal and pathological intestinal conditions. To achieve this, we conducted repeated testing of lactulose, mannitol and GIP excretion dynamics in a cohort of 12 healthy volunteers under fasting conditions, performing the test three times per patient, to assess intraindividual variability and the consistency of GIP as a biomarker compared with the LMR.

Although the volunteers were instructed to adhere to a gluten-free diet and to avoid both mannitol and lactulose throughout the study, each individual was otherwise free to follow their regular diet, introducing a degree of natural variability into the study design reflecting real-life conditions. Thus, our study not only evaluates intraindividual consistency under standardized conditions but also examines the behavior of these biomarkers in response to moderate dietary changes, such as those commonly occurring in daily life. This strengthens the interpretation of our findings by demonstrating that, even under less controlled conditions, the biomarkers studied, particularly GIP, exhibit notable robustness and reliability.

The results of the current study revealed similar excretion patterns for u-GIP, lactulose and mannitol. Similar to the results of a previous study (13), double excretion peaks were observed in some volunteers, suggesting variability in absorption and excretion. This suggests the presence of individual, reproducible characteristics that influence the lactulose absorption and excretion dynamics. A possible explanation could be regional differences in intestinal permeability, where certain areas, depending on their orientation (vertical vs. horizontal) or anatomical structure, may exhibit distinct absorption properties for lactulose. Variations in transit time, motility and segment-specific permeability could contribute to this phenomenon, highlighting the complexity of lactulose excretion patterns (8, 19). For mannitol, the results were also consistent between studies, showing the absence of complete excretion in all volunteers, likely due to the difficulty of following a diet completely free of mannitol contributions.

As shown in **Table 2**, a few outlier values were observed in some volunteers for different parameters. However, it is important to note that no volunteer showed outlier values for any parameter across all three test days. These values were excluded from the analysis of intraindividual variability, which increased the reliability and robustness of the results. This exclusion helps to establish a normal biological pattern without the influence of extreme or atypical data points. It is worth noting that the exclusion of outlier values is a recognized statistical practice that helps minimize the impact of potential experimental errors, technical variability, or unusual physiological responses that do not reflect the overall behavior of the sample. By removing these isolated deviations, the analysis more accurately captures the central tendency and reproducibility of the measurements under normal physiological conditions (20).

According to the data presented in **Figure 2**, LMR showed the greatest number of deviations from the reference range. Among the results of the two different time intervals for the LMR, the 0–15-hours interval showed higher similarity to the findings of an earlier investigation. In contrast, nearly all GIP results in this study fell within the reference range established in our prior research across both time intervals, reflecting a higher level of reproducibility than the LMR. This discrepancy in the LMR values suggests increased biological variability or different physiological responses to the administered compounds. Since the LMR is calculated from the absorption of non-metabolizable sugars, whereas GIP measurements are based on peptide excretion, this variability may stem from differences in transport and metabolic pathways between sugars and peptides. Therefore, the consistency of the GIP results strengthens its potential as a reliable biomarker, because reduced variability enhances its applicability in clinical settings. Additionally, the higher reproducibility observed in the 0–15-hours interval for the LMR compared to the 0–6-hours interval reinforces the conclusions drawn in our previous study (13), indicating that a shorter interval may miss a substantial proportion of excretable probes that may generate higher variability than collecting longer periods of the excreted probes, in contrast to the conventional approach typically used in such tests (14). Notably, urinary excretion patterns varied considerably between individuals; for instance, in the present study, one participant did not produce any urine until 8 hours after ingestion, meaning all excretion data from the 0–6-hours interval would have been missed. This highlights the limitations of short collection windows and supports the need for extended sampling to ensure accurate and representative measurements. The LMR is often not recommended as an analytical marker of the intestinal function in clinical studies because of its high variability. Extending the urine collection period to 9–15-hours may also account for additional physiological processes, such as individual differences in digestion time, intestinal transit and metabolic clearance, which are not fully captured within the conventional 0–6-hour window (14).

**Figures 3a, b** presents the CV for the different parameters analyzed at 0–6 and 0–15-hour intervals. As shown in the figure, the average CV for the four parameters remained approximately 20%, which is considered acceptable given the study conditions (including repeated measurements on different days and dietary variations) (21). Moreover, the maximum CV did not exceed 40% in any case, with the sole exception of mannitol in the 0–6-hour interval, in which some volunteers reached values close to 50%, as reflected by the length of the boxplot whiskers. These results suggest that despite individual variability, the intraindividual reproducibility of the measurements was adequate and the analyzed parameters exhibited consistent behavior throughout the study.

The ICC is a statistical indicator that assesses the consistency of measurements within the same individual compared with variations between individuals. As shown in **Table 4**, in the case of GIP excretion, the ICC decreased from 0.61 to 0.32 upon removing outliers. This decrease suggests that the presence of outliers leads to a perception of lower intraindividual variability than interindividual variability, resulting in a high ICC. Upon removing these outliers, the data became more closely grouped, which reduced the interindividual variability. Although intraindividual values became more consistent after outlier removal, the differentiation between data from different individuals became less evident. Thus, although the data initially presented a high ICC, indicating that the measurements were consistent within each individual and distinct between individuals, the total variability ceased to be primarily attributable to the differences between individuals after the outliers were removed, leading to a reduction in the ICC. This outcome is positive because all individuals belong to the same group; therefore, having data that are consistent at both the intraindividual and interindividual levels is ideal.

Conversely, for the LMR measured in the 0–6-hour interval, the ICC increased from 0.28 to 0.47 after excluding outliers. This increase indicated that the initial low ICC was due to a lack of intraindividual consistency, influenced by the presence of outliers. This introduced significant variability among the individuals, masking the true consistency of the measurements. By removing the outliers, the measurements within each individual across the three study days became more consistent, resulting in a higher ICC. This finding underscores the importance of outlier removal to enhance measurement reliability, revealing clearer patterns of individual reproducibility over time. Compared with the study conducted by Kuzma et al. (17), in which the LMR test showed “fair” reliability with an ICC of 0.53, our results indicated that despite a slightly lower ICC of 0.47, both results are similar and suggest moderate consistency in the measurements. Furthermore, similar to their study, we consider that the ICC for the LMR could be higher if our study included individuals with certain gastrointestinal disorders associated with a substantially elevated LMR.

The ICC for GIP increased to 0.75 in the 0–15-hour interval, indicating that longer collection times improve both the consistency within individuals and the ability to distinguish between them. This suggests that GIP excretion stabilize over time, making individual patterns clearer while remaining reproducible. This reinforces the value of GIP as a reliable biomarker for capturing interindividual differences in a healthy population.

In summary, the intraindividual variability analyses (reference ranges, intraindividual CV and ICC) conducted in this study provided a comprehensive perspective on the reliability of the measurements for the different parameters evaluated. The results for the GIP and LMR suggest that while both markers are informative, GIP shows greater reproducibility. The GIP demonstrated high stability, with most of its values falling within the reference range, acceptable CV and an ICC reflecting good intraindividual consistency. Thus, the GIP are a robust and reliable biomarker with promising clinical potential. In comparison, the LMR, although still useful and widely employed as a reference (11), exhibited higher variability (9), with more values outside the reference range and a low initial ICC, which only improved after the exclusion of outliers. This indicates that its reliability may depend on better control of experimental variables. Thus, although the LMR remains a widely used reference, the GIP may provide greater stability for future applications.

When comparing the cumulative excretion results for the 0–6-hour interval against the total excretion over the 0–15-hour period, we observed slight differences between our previous and current studies. In the first study, the cumulative excretion percentages were approximately 55%, 73% and 56% for lactulose, mannitol and GIP, respectively (13). In the present study, these percentages increased to approximately 70%, 77% and 62% for lactulose, mannitol and GIP, respectively. The most notable change was the increase in lactulose excretion from 55% to 70%. Lactulose has variable excretion patterns. Because of this variability, it was essential to normalize the lactulose results using mannitol as a reference, as the latter helps to account for fluctuations in lactulose absorption as has been described by other authors (22). Therefore, the differences in lactulose excretion between the two studies may have been influenced by microbiome interactions or other individual features.

This study had some limitations, such as the sample size, which consisted of only 12 healthy volunteers, potentially affecting the generalization of the results and the absence of patients with diagnosed gastrointestinal conditions related to gut permeability such as celiac disease, irritable bowel syndrome or inflammatory bowel disease. This limits the immediate applicability of the results to clinical populations and future studies in these groups will be essential to assess the method’s clinical relevance and performance. In this regard, independent validation by external research groups will be crucial to confirm the utility and reproducibility of the u-GIP method across different clinical settings. Additional studies are already being conducted in collaboration with hospitals and academic institutions to further support its clinical translation. Nonetheless, the repetition of tests over three days for each volunteer provides a necessary attempt to evaluate the intraindividual variability to establish reference data for normal gut permeability. Furthermore, the fact that the participants did not need to follow a strictly uniform diet during the tests allowed us to verify the robustness of the method, considering the future practice in clinical settings. This work is, to our knowledge, the first study to implement this approach. However, it would be ideal to conduct a test in individuals with gastrointestinal disorders associated with increased paracellular intestinal permeability to evaluate the effectiveness of this methodology in a clinical context. In addition, further research is needed to assess the potential applicability and safety of the u-GIP method in other age groups, such as children and the elderly. Age-related physiological differences—such as changes in intestinal permeability, renal clearance and immune response—may influence GIP absorption and excretion. Therefore, specific studies in these populations are essential before considering clinical implementation. Although u-GIP may serve as a robust biomarker, its clinical use in individuals with gluten sensitivity or allergy requires careful consideration. The administration of gluten in these populations may pose potential risks and could influence data interpretation. However, several studies (23–26) have conducted controlled gluten challenge protocols in both celiac patients and individuals with non-celiac gluten sensitivity, providing a basis for the safe and ethical evaluation of gluten exposure in sensitive populations under medical supervision. These precedents support the feasibility of applying the u-GIP method in such contexts, provided that appropriate protocols and safeguards are implemented. Before proceeding, it would be beneficial to optimize certain aspects of the protocol, such as the amount of gluten to be ingested; 10 g may not be the most comfortable or suitable dose for individuals with gluten sensitive conditions. Finally, it is crucial to conduct further studies in patients with abnormal gut permeability to assess the real potential and obtain satisfactory sensitivity and specificity of the method for the detection and monitoring of paracellular intestinal hyperpermeability. In this regard, the use of u-GIP as permeability markers presents an opportunity to improve current testing strategies. Compared to traditional probes such as lactulose and mannitol, u-GIP offer distinct advantages, including their direct clinical relevance as immunogenic dietary proteins, the ability to detect gluten translocation without the need for normalization, reduced physiological variability and compatibility with fast, non-invasive methods such as LFIA. Moreover, lactulose may induce undesirable gastrointestinal symptoms such as bloating, flatulence, abdominal discomfort, or diarrhea, which can limit its applicability in sensitive populations and confound clinical assessments (12).

## Data Availability

The raw data supporting the conclusions of this article will be made available by the authors, without undue reservation.
